# RNA-seq data of banana bunchy top virus (BBTV) viruliferous and non-viruliferous banana aphid (*Pentalonia nigronervosa*)

**DOI:** 10.1016/j.dib.2019.104860

**Published:** 2019-11-21

**Authors:** Siti Subandiyah, Ruth Feti Rahayuniati, Sedyo Hartono, Susamto Somowiyarjo, Afiahayati, Alan Soffan

**Affiliations:** a Department of Plant Protection, Faculty of Agriculture, Universitas Gadjah Mada, Yogyakarta, Indonesia; b Research Center for Biotechnology, Universitas Gadjah Mada, Yogyakarta, Indonesia; c Department of Computer Science and Electronics Faculty of Mathematics and Natural Sciences Universitas Gadjah Mada, Yogyakarta, Indonesia; d Department of Agrotechnology, Faculty of Agriculture, Universitas Jenderal Soedirman, Purwokerto, Indonesia

**Keywords:** Banana aphid, BBTV, RNA-Seq, Transcriptomic analysis

## Abstract

Banana bunchy top disease (BBT) is one of the most economically serious viral diseases of banana caused by banana bunchy top virus (BBTV: Nanoviridae: Babuvirus). BBTV is a circular, ssDNA virus which is suitable in the phloem tissue and currently only being transmitted by the banana aphid (*Pentalonia nigronervosa*) in a persistent, non-propagative, circulative manner. Interaction of BBTV and banana aphid had been studied in several ways, such as transmission and translocation of BBTV inside the banana aphid body at cellular level. However, the molecular mechanism underlying the interaction between BBTV and banana aphid have been poorly understood. Therefore, this transcriptomic study was conducted to obtain the raw data for differential genes expression study in BBTV viruliferous (Vr) and non-viruliferous (NVr) banana aphid. Here, we present two data sets of RNA seq raw reads which is available in GenBank Sequence Read Archive (SRA) database with accession number of SRX6918251 and SRX6918252 for the Vr and NVr banana aphid respectively.

**Table:** Specifications Table

Subject	Insect Science
Specific subject area	Transcriptomics
Type of data	Transcriptome sequences (RNA-Seq raw reads)
How data were acquired	BGISEQ-500 sequencing platform
Data format	Raw sequences (FASTQ)
Parameters for data collection	BBTV viruliferous (Vr) and non-viruliferous (NVr) of banana aphid, *Pentalonia nigronervosa*
Description of data collection	Vr and NVr banana aphid samples (whole body) were collected from BBTV infected and non-infected banana plant respectively. Total RNA was isolated and cDNA libraries were prepared for RNA-sequencing. The RNA-seq raw reads were further analyzed to get the clean reads and stored in FASTQ file.
Data source location	Universitas Gadjah Mada, Yogyakarta, Indonesia, GPS data: 7.768721, 110.381900
Data accessibility	Raw data (FASTQ) of Vr and NVr banana aphid has been deposited in NCBI Sequence Read Archive (SRA) data base with the accession numbers SRX6918251 and SRX6918252 respectively.

**Table:** 

**Value of the Data** • BBTV is one of the most economically serious viral diseases of banana, transmitted by the banana aphid, in a persistent, non-propagative, circulative manner [[1], [2], [3], [4]]. • Two set of raw-FASTQ files transcriptome data, Vr and NVr banana aphids, were reported here to support the understanding of molecular mechanism underlying the interaction of BBTV and the insect vector, banana aphid. • The presented data can be further analyzed by performing differential gene expression study which is essential in determining the majors genes involved in the BBTV transmission success by banana aphid vector, which finally may support BBTV management.

## 1 Data

FASTQ raw data file which was generated from two sets of Vr and NVr banana aphid transcriptome has been deposited to NCBI-SRA data base with the accession number SRX6918251 and SRX6918252 respectively. Methods of insect rearing and collection, total RNA extraction, sequencing and generating clean transcriptome data is presented in the following section.

## 2 Experimental design, materials, and methods

### 2.1 Insect rearing, sample collection and BBTV detection

Initial colony of banana aphids were obtained from banana plantation grown in Bantul, Yogyakarta, Indonesia, and further transferred to the greenhouse facilities in Universitas Gadjah Mada, Yogyakarta. BBTV infected and non-infected banana were utilized for the rearing of BBTV Vr and NVr banana aphid respectively. New banana seedlings were continuously provided to replace old banana seedling to keep banana aphid population exist. Pool of 40 individuals banana aphid at different instars were collected and immersed on RNA later (Ambion) for RNA extraction. Confirmation of the BBTV viruliferous banana aphid population was conducted by amplifying the BBTV primers BBT1: 5′-CTCGTCATGTGCAAGGTTATGTCG-3′ and BBT 2: 5′-GAAGTTCTCCAGCTATTCATCGCC-3′, on pool of 10 adult aphid DNA (Geneaid DNA extraction kit) targeting 250–350 bp PCR product [5].

### 2.2 RNA isolation, library preparation and RNA-seq

Both Vr and NVr banana aphid samples (whole body) were RNA extracted using RNeasy Plus kit (Qiagen, MD, USA) according to the manufacturer's instructions. The quantity and quality of the total RNA were validated using NanoDrop spectrophotometer (Thermos, USA) for the purity of the RNA samples, and Agilent 2100 Bioanalyzer (Agilent RNA 6000 Nano Kit) for the RNA integrity (RIN value), 28S/18S and the fragment length distribution. The samples were further sequenced using BGISEQ-500 platform following the steps as follow; 1) mRNA enrichment, 2) RNA fragment and reverse transcription: 3) End repair, 4) PCR amplification, 5) Denature and cyclization, 6) Sequencing on BGISEQ- 500 platform.

### 2.3 RNA-seq data workflow

Filtering step was first performed on the raw sequencing reads generated by RNA-seq., mainly by removing those raw reads with adaptors and reads with more than 5% of unknown bases (N). After filtering, the remaining reads are called “Clean Reads” and stored in FASTQ file. Those clean read then ready for further assembly process. Descriptive statistics on the RNA-seq data of the two set of both Vr and NVr banana aphid samples are given in Table 1.

**Table 1:** Descriptive information for RNA seq raw data for two samples of BBTV viruliferous (Vr) and non-viruliferous (NVr) banana aphid.

Descriptive	Sample	
	Nvr	Vr
Total Raw Reads (Mb)	69.73	69.73
Total Clean Reads (Mb)	67.09	67.02
Total Clean Bases (Gb)	6.71	6.7
Clean Reads Q20 (%)	98.83	98.85
Clean Reads Q30 (%)	93.47	93.6
Clean Reads Ratio (%)	96.22	96.12
Biosample ID	SAMN12868030	SAMN12868029

Total Raw Reads(Mb):The reads amount before filtering.

Total Clean Reads(Mb):The reads amount after filtering.

Total Clean Bases(Gb):The total base amount after filtering.

Clean Reads Q20(%):The rate of bases which quality is greater than 20 value in clean reads.

Clean Reads Q30(%):The rate of bases which quality is greater than 30 value in clean reads.

Clean Reads Ratio(%):The ratio of the amount of clean reads.
